# Plasma volume expansion from the intravenous glucose tolerance test before and after hip replacement surgery

**DOI:** 10.1186/1742-4682-10-48

**Published:** 2013-08-26

**Authors:** Robert G Hahn, Thomas Nyström, Stefan Ljunggren

**Affiliations:** 1 Department of Anesthesia, Faculty of Health Sciences, Linköping University, Linköping, Sweden; 2 Research Unit, Södertälje Hospital, Södertälje, 152 86, Sweden; 3 Department of Clinical Science and Education, Södersjukhuset, Karolinska Institutet, Stockholm, Sweden

**Keywords:** Kinetic model, Intravenous glucose tolerance test, Plasma volume expansion

## Abstract

**Background:**

Hyperosmotic glucose is injected intravenously when an intravenous glucose tolerance test (IVGTT) is initiated. The extent and time period of plasma volume expansion that occurs in response to the glucose load has not been studied in the perioperative setting.

**Methods:**

Twenty-two non-diabetic patients aged between 57 and 76 years (mean 68) underwent an IVGTT, during which 0.3 g/kg of glucose 30% (1 ml/kg) was injected as a bolus over one minute, one day before and two days after hip replacement surgery. Twelve blood samples were collected over 75 minutes from each patient. The turnover of both the exogenous glucose and the injected fluid volume was calculated by means of mass balance and volume kinetic analysis.

**Results:**

The IVGTT raised plasma glucose by 9 mmol/L and the plasma volume by 8%. The extracellular fluid volume increased by 320 (SD 60) ml of which 2/3 could be accounted for in the plasma. The half-life of the exogenous glucose averaged 30 minutes before surgery and 36 minutes postoperatively (*P* < 0.02). The glucose elimination governed 86% of the decay of the plasma volume expansion, which occurred with a half-life of 12 minutes before to 21 minutes after the surgery (median, *P* < 0.001).

**Conclusion:**

Hyperosmotic glucose translocated intracellular water to the plasma volume rather than to the entire extracellular fluid volume. The preferential re-distribution acts to dilute the plasma concentrations used to quantify insulin sensitivity and ß-cell function from an IVGTT. The greater-than-expected plasma dilution lasted longer after than before surgery.

## Background

The intravenous glucose tolerance test (IVGTT) is a tool for the assessment of insulin sensitivity and ß-cell function in diabetes research [1,2] and perioperative studies [3,4]. A bolus injection of glucose is given and the plasma concentrations of glucose and insulin are usually measured over three hours. The data can be analyzed by, for example, the minimal model [5] and the single delay model [6]. The IVGTT requires frequent blood sampling and is labor-intensive. Therefore, the authors have developed a 75-minute IVGTT that is more practicable to apply in the perioperative setting [3].

Another problem is that the IVGTT is likely to cause a sudden increase of the plasma volume. Although the injected fluid volume is small (60–80 ml), the glucose solution has traditionally six times the osmotic strength of body fluids. The hypertonicity translocates water from the intracellular (ICF) to the extracellular fluid (ECF) space, where it augments the plasma volume expansion [7]. The magnitude of the plasma volume expansion resulting from an IVGTT over time has previously been studied in young, healthy volunteers [8], although the test is usually performed in elderly patients. Moreover, plasma volume kinetics has not been studied in the perioperative setting.

In the present study, the volume kinetic models developed for hypertonic fluids [7,9] and glucose [10,11] were combined to analyze the disposition of the injected test solution during IVGTT in 22 patients before and after hip replacement surgery. The reason why two models have to be combined is that the hypertonic glucose is a driving force for body-fluid shifts. Hip replacement is performed in senior citizens and the comparison of pre- and postoperative data illustrates the effect of surgical stress on glucose turnover and its subsequent influence on the plasma volume.

## Patients and methods

### Patients

Twenty-two non-diabetic patients, 15 females and seven males, aged between 57 and 76 years (mean 68), and with a body weight of 46–101 kg (mean 80), were studied before and after undergoing elective total hip replacement surgery at the Orthopedic Departments at Södersjukhuset, Stockholm and Södertälje Hospital, Södertälje. The study was approved by the Regional Ethics Committee of Stockholm (Ref. 2011/1141-31/3). Each patient gave written consent for participation.

### Intravenous glucose tolerance test

An IVGTT was performed to estimate the insulin sensitivity of each participant. The patients arrived at the laboratory at 8 AM on the day before their surgery and two days after their surgery. On both occasions, the patients had fasted since midnight. An intravenous catheter was inserted into an antecubital vein of one arm, for infusion of insulin and glucose, respectively. Another cannula used for blood sampling was inserted into an antecubital vein of the opposite arm.

After a 30-minute equilibration period to obtain hemodynamic steady state, a short regular IVGTT was performed by administrating 0.3 g/kg of glucose in a 30% solution over one minute. Blood was sampled from the contralateral antecubital vein at the 0, 2, 4, 6, 8, 10, 20, 30, 40, 50, 60 and 75-minute intervals for measurement of the plasma glucose and blood hemoglobin (Hb) concentrations. Plasma glucose was measured by the glucose oxidase method (Modular P, Roche Diagnostics, Tokyo, Japan) and Hb by colorimetry (Technicon Advia, Bayer, Tarrytown, NY, USA), at the certified clinical chemistry laboratory at the Karolinska University Hospital.

### Pharmacokinetics

#### Glucose

Plasma concentration *G* at time *t* when glucose was infused at rate *R*_o_ was calculated using the following differential equation:

$$\frac{d\left(G-G_{b}\right)}{\mathrm{dt}}=\frac{R_{o}}{V_{d}}-\frac{\mathrm{CL}}{V_{d}}\left(G\left(t\right)-G_{b}\right)$$

where G_b_ is the baseline glucose, *V*_d_ is the volume of distribution, and *CL* is the clearance. Removal of glucose from *V*_d_ corresponds, in the absence of glucosuria, to the uptake of glucose by cells [10,11]. As very high concentrations of glucose (“overshoot”) could sometimes be detected just after the injection, all kinetic analyses disregarded the data points between two and eight minutes.

#### Fluid

Hypertonic glucose translocates water from the intracellular (40% of body weight [BW]) to the extracellular (20% of BW) fluid space in proportion to the added amount of osmotic active molecules and the accompanying water volume. The translocated volume *f*_t_, resulting from the injection, was obtained by the following mass balance calculation which was based on the induced deviation from the baseline osmolality in all body fluids, which is 295 mosmol/kg [7]:

$$\;\frac{\mathrm{BW}\cdot 20\%\cdot 295+\mathrm{infused}\;\mathrm{osmoles}}{\mathrm{BW}\cdot 20\%+f_{t}+\mathrm{infused}\;\mathrm{volume}}=\frac{\mathrm{BW}\cdot 40\%\cdot 295}{\mathrm{BW}\cdot 40\%-f_{t}}$$

The calculated value of *f*_t_ is then inserted into a one-volume kinetic model [10], which calculated the baseline volume of distribution (*V*) and the clearance (*CL*) for the sum of the injected (*R*_*o*_) and translocated (*f*_t_) fluid volumes. In this kinetic model, fluid added to the kinetic system expands *V* to *v*, which strives to return to *V* by the elimination of fluid at a rate proportional to a constant *CL* of the dilution of *V*, and second, a baseline loss (*CL*_*o*_) fixed to 0.4 ml/min to account for evaporation and basal diuresis. The volume change of *v* is then expressed as [12]:

$$\frac{\mathrm{dv}}{\mathrm{dt}}=R_{o}+f_{t}-{\mathrm{CL}}_{o}-\mathrm{CL}\frac{\left(v-V\right)}{V}$$

The dilution of *v* is given by (*v*-*V*)/*V* and was equal to the plasma dilution as derived from the blood Hb concentration at baseline (time 0) and at a later time *t*. Hence: [(Hb_o_/Hb(t))–1]/(1–hematocrit_o_). While *V* is a functional body fluid space, tracer methods support that the Hb-derived plasma dilution can be used to accurately infer the percentage expansion of the plasma volume [13-15].

The optimal estimates for the unknown parameters in the glucose (V_d_ and *CL*) and fluid models (V and *CL*) were calculated for each of the 22 experiments individually through nonlinear least-squares regression based on a modified Gauss-Newton method. The input variables were plasma glucose, plasma dilution, and *t.* No weights were used. The software was Matlab 4.2 (Math Works Inc., Natick, MA, USA).

The half-life (T_1/2_) of the glucose load was given by [ln 2 *V*_d_ / *CL*] while T_1/2_ of the induced plasma volume expansion was taken as [ln 2 *V* / *CL*].

### Statistics

The results were presented as mean, standard deviation (SD) and, when there was skewed distribution, as the median (25^th^-75^th^ percentile range). Between-patient variability of plasma glucose and plasma dilution was calculated as the absolute residual error between each individual measurement and time-matched modeled data based on the median optimal parameter estimates for the group, as shown in the Table 1. Differences between pre- and postoperative data were compared by the Wilcoxon’s matched-pair test, using SPSS version 20 (IBM 2011). *P* < 0.05 was considered statistically significant.

**Table 1:** Baseline and kinetic data for injected glucose and fluid during an intravenous glucose tolerance test before and after hip replacement surgery in 22 patients

	**Before surgery**	**After surgery**	**Statistics**
Plasma glucose, baseline (mmol/L)	5.6 (5.3-5.8)	6.0 (5.8-6.5)	*P* < 0.001
B-Hb concentration (g/dL)	12.4 (12.0-13.3)	10.0 (9.0-10.6)	*P* < 0.001
***Glucose kinetics***			
V_d_ (L)	12.7 (10.2-15.6)	14.0 (12.1-14.8)	NS
*CL* (ml/min)	325 (272–385)	260 (220–306)	*P* < 0.02
T_1/2_ (min)	30 (19–34)	36 (30–45)	*P* < 0.02
***Fluid kinetics, no glucose uptake***			
V (L)	2.94 (2.17-3.60)	2.83 (2.62-3.22)	NS
*CL* (ml/min)	200 (70–261)	93 (64–189)	*P* < 0 001
T_1/2_ (min)	12 (5–30)	21 (10–33)	*P* < 0.001
***Fluid kinetics, with glucose uptake***			
V (L)	2.79 (2.50-3.42)	2.73 (2.40-3.05)	NS
*CL* (ml/min)	37 (−7 to 165)	9 (−14 to 55)	*P* < 0.02

Data is the median and 25^th^-75^th^ percentile limits.

The Wilcoxon matched-pair test was used for statistics. NS = not statistically significant.

## Results

The amount of injected glucose solution was 80 (SD 14) ml. Several patients reported transient (1–2 min) warmth from the injection site in the arm to the face and legs, and occasionally an unpleasant feeling in the palate region.

**Figure 1:**
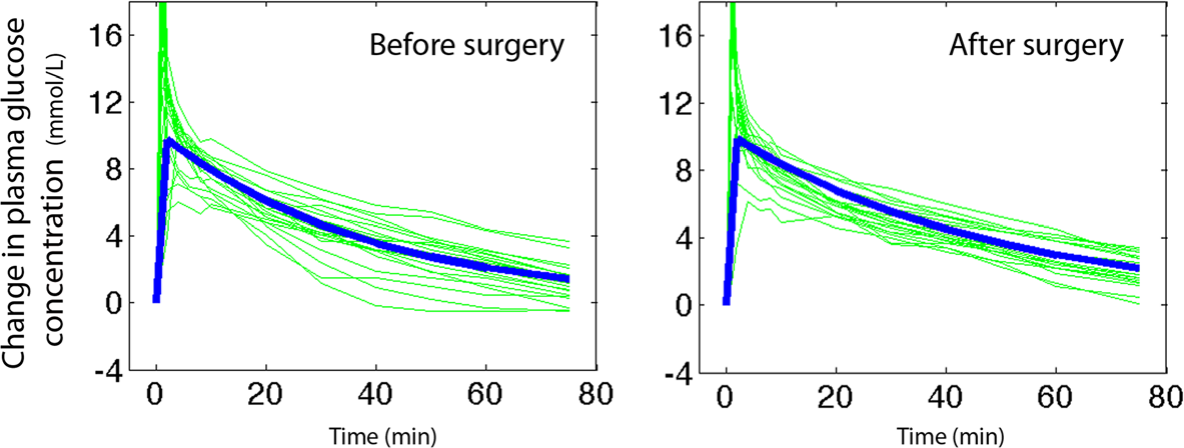
**The increase in plasma glucose induced by an intravenous glucose tolerance test (IVGTT), consisting of an intravenous injection of 0.3 g/kg of glucose over 1 minute, performed before and after hip replacement surgery.** Each experiment is represented by a thin line and the modeled average by a thick line.

The baseline plasma glucose concentration was 7% higher and Hb was 19% lower after surgery than before (Table, top). On both occasions, the modeled rise in plasma glucose was 9 mmol/L (Figure 1). The surgery increased T_1/2_ of the glucose by 20%, which was due to a reduction of *CL* while *V*_d_ remained essentially constant (Table, middle). Surgery also reduced between-patient variability of plasma glucose by 18% (*P* < 0.012).

The injection of a 30% glucose solution translocated water from the ICF space, which was calculated by mass balance to be 1.82 ml for each mmol of injected glucose (=f_t_). As the dose was 0.3 g/kg, the amount of translocated fluid could be estimated to be 241 (45) ml. Thus, the sum of the injected and translocated fluid volumes would theoretically amount to 320 (60) ml.

**Figure 2:**
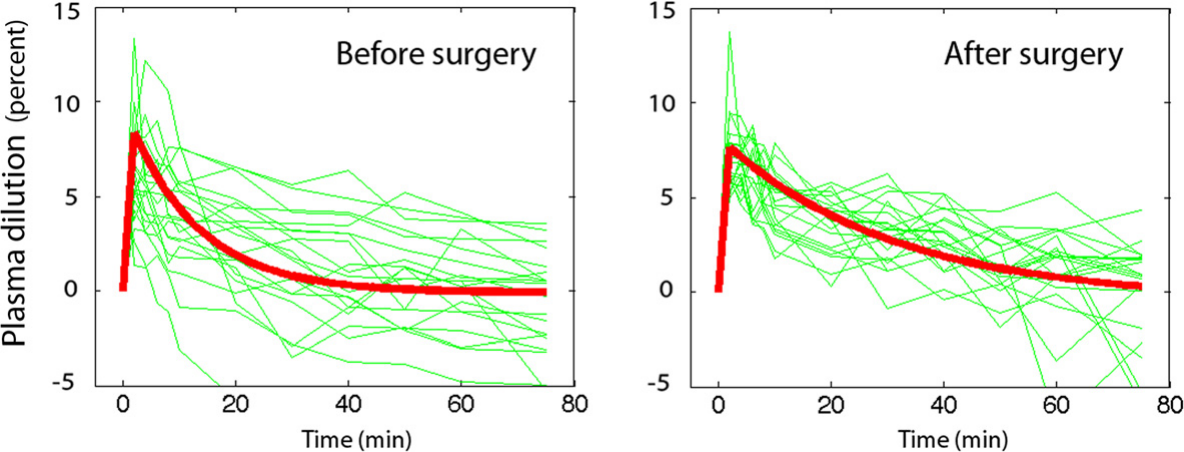
**The dilution of venous plasma in response to an IVGTT performed before and after hip replacement surgery.** Each experiment is represented by a thin line and the modeled average by a thick line.

Most of this volume could be found in the intravascular compartment. The measured maximum dilution of venous plasma amounted to 8% (Figure 2) and the volume kinetic analysis showed that the fluid expanded an ECF space (*V*) of 2.9 (0.9) L, with little difference depending on whether the kinetic analysis was based on data obtained before or after surgery (Table). The maximum plasma volume expansion, which equals product of plasma dilution and *V*, would then average 230 ml. Hence, two-thirds of the sum of injected and translocated ICF volume could be accounted for in the plasma.

The rate of decay of the plasma volume expansion occurred more slowly after surgery than before (Figure 2). The surgery also decreased the between-patient variability of the plasma dilution by 32% (*P* < 0.001). The fluid *CL* was only half as high, which almost doubled T_1/2_ for the volume expansion_,_ from 12 minutes to 21 minutes (Table, bottom).

The fluid *CL* became reduced by 85% (median) if account was taken of the water volume that was osmotically re-translocated to the cells along with the uptake of glucose was disregarded (Table, bottom). Some *CL* values even became negative, which made the authors refrain from calculating T_1/2_.

## Discussion

The clinician should be aware that injecting hypertonic glucose during an IVGTT induces an abrupt expansion of the plasma volume in all patients. Fortunately, the magnitude in elderly surgical patients is not greater than in younger healthy volunteers [8], but the hemodynamic consequences are naturally dependent on the cardiac reserves of the subject undergoing the test. For example, the volume load could elicit acute decompensation in patients with worsening heart failure. We recommend that clinicians be prepared to administer a vasodilator drug intravenously if performing an IVGTT in such patients.

The volume kinetic calculations showed that the hypertonic glucose increased the plasma volume by 230 ml within 2 minutes. This volume was obtained by multiplying the maximum Hb-derived plasma dilution with the size of *V*. The latter is a functional body fluid space but can be regarded to equal the plasma volume when the size is slightly smaller than an estimate based on anthropometric measures: the blood volume in these patients can be assumed to equal 7% of the body weight and the baseline plasma volume, with regard to the hematocrit, would then be 3.6 L. Using the theoretical 3.6 L as the basis for the calculation the maximum plasma volume expansion would be 288 ml. The difference from 230 ml might be due to the old age of the studied patients, but could also be within the range of errors inherent in the methods used and the assumptions made in anthropometric estimates. The total expansion of the ECF volume based on mass balance was estimated to be only slightly larger, 320 ml.

These calculations illustrate that the intravascular volume expansion was disproportionately large based on the belief that osmosis-driven translocation of water from the ICF distributes equally throughout the ECF space. As seen in several previous studies, the size of body fluid space expanded by fluid (*V*) is remarkably low when being a part of glucose administration. In fact, V is more consistent with the plasma volume (here, 2.7-2.9 L) than with the size of the ECF space [8,10,16]. This is surprising, as the volume of distribution for the exogenous glucose (*V*_d_) was four times larger. The difference between *V*_d_ and *V* might reflect higher compliance for glucose molecules than for water in penetrating and expanding the jelly matrix of the extravascular part of the ECF space. However, this explanation is less likely as salt-containing isotonic and hypertonic infusion fluids do expand extravascular parts of the ECF volume [7,9]. In any event, to maintain osmotic equilibrium the difference between *V* and *V*_d_ assumes that most of the injected glucose is exchanged for electrolyte-rich fluid at the border of the capillary membrane.

How glucose affects fluid turnover is illustrated by the data on fluid elimination. After the acute volume expansion, most of the excess fluid volume residing in the plasma was eliminated by virtue of osmosis. Therefore, the T_1/2_ of glucose is apparently a key factor for the duration of the plasma volume expansion following an IVGTT. Previous studies of young healthy volunteers have yielded a T_1/2_ for glucose of between 11 minutes and 15 minutes [8,10], while during laparoscopic surgery in females at 40 years of age T_1/2_ was 30 minutes [16]. The patients in the present study were almost 30 years older and had a T_1/2_ of 30 minutes even before the surgical stress, which suggests that plasma volume expansion after an IVGTT becomes more long-lasting due to both age and surgery. The slower turnover of glucose after surgery is mainly caused by reduced insulin sensitivity, which drops by approximately 35-50% after hip replacement [4]. Surgery also reduced the between-patient variability of plasma glucose and Hb dilution, which was probably due to alignment to similar degrees of the vascular tonus and degree of “stress” postoperatively.

Glucose uptake did not account for the entire reduction of the plasma volume expansion after the IVGTT was initiated. A residual reduction, which was apparently due to non-osmotic mechanisms, could be estimated by accounting for the effect of glucose uptake on the decay of the volume expansion, and amounted to 15% of the total. This is shown by the lower *CL* of fluid obtained when the calculations accounted for uptake of glucose to the cells (Table, bottom). Renal excretion is the most likely mechanism for such non-osmotic elimination, which is known to operate more slowly during the postoperative phase, because of the influence of water-retaining “stress” hormones such as cortisol and aldosterone [17]. However, as only a small fraction of the fluid was eliminated by non-osmotic mechanisms, the calculated elimination approached and even passed zero elimination in some patients. This shows that the accuracy of the methods and the assumptions made were not always capable of capturing fluid shifts amounting to 20–30 ml. On the other hand, all residual eliminations would have been positive if the baseline fluid loss of 30 ml (*CL*_o_) had not been deducted from plasma volume expansion.

The clinical importance of the present calculations consists in the demonstration of an abrupt increase of the plasma volume expansion that occurs when an IVGTT is initiated. The expansion is greater than would be expected based on belief that the water translocated from the ICF by hypertonic glucose is evenly distributed throughout the ECF volume. The small *V*_d_ for the injected and translocated fluid volumes means that hypertonic glucose is approximately three times more effective than hypertonic saline to expand the plasma volume [7,9,18]. The volume expansion obtained from 30% glucose was also 10 times greater than for Ringer’s acetate solution infused over 30 min in the elderly [17]. The volume expansion will also last much longer if the subject is in a state of insulin resistance, such as during the postoperative period in senior citizens.

A scientific concern raised by the present study is that the plasma dilution distorts the relationship between amount and concentration of any substance measured in plasma sampled during an IVGTT. The plasma is likely to show more erroneously low concentrations the earlier the IVGTT experiment sampling is made. For example, plasma insulin sampled early during an IVGTT will show a 8% lower concentration compared to insulin alone being injected. Twenty minutes later, when the plasma volume expansion is only 4%, the distortion will be only half as great. These effects act to overestimate *CL* for glucose, while T_1/2_ becomes underestimated compared to calculations based on amounts. These considerations are probably trivial in most instances. However, more complex kinetic calculations based on IVGTT, such as the minimal model [5], single delay model [6] and deconvolution of C-peptide concentration [19], are likely to be affected. These effects are not easy to predict or reconstruct with confidence, since the variability of the plasma dilution response to IVGTT is greater than the variability of the plasma glucose response (cf. Figures 1 and 2). However, for accurate results with regard to amounts the input values in kinetic calculations should be corrected by dividing all concentrations by the plasma dilution. The authors wish to quantify these effects in subsequent works. Staggered by the strong plasma volume expansion reported here the authors also plan to compare the plasma volume expansion resulting from hypertonic glucose with that obtained by small amounts of hypertonic saline and other fluids.

Limitations of the present study include that the plots of plasma glucose over time (Figure 1) show that an “overshoot” frequently occurred within the first minutes after an IVGTT was initiated. The plasma glucose concentration was then transiently higher than expected from fitting a one-compartment model to the data, but the authors refrained from applying a two-compartment model as the “overshoot” was inconsistent and the data points too few to allow detailed analysis. While this “overshoot” could affect fluid distribution during the first minutes of the study, the curve-fitting routine was set to disregard the plasma glucose concentrations measured between 2 and 8 min of the experiments. These data were also omitted when the between-patient variability in plasma glucose and Hb dilution was calculated.

Another limitation of this study was that diabetic patients were not included. As the fluid clearance is governed almost completely by the rate of uptake of glucose in the cells, patients with diabetes are likely to have even more long-lasting plasma volume expansion than the postoperative patients studied here. A final limitation of the present study was that no hemodynamic measurements were made, and these could have improved the presentation.

## Conclusion

The acute plasma volume expansion resulting from an IVGTT amounted to 8% both before and after hip replacement surgery. The duration of the plasma volume expansion was twice as long postoperatively than before the surgery. This effect was mostly attributable to slower turnover of the injected glucose. For accurate results, calculations based on an IVGTT should be corrected for plasma dilution induced by the hypertonic glucose.

## Abbreviations

AUC: Area under the curve; CL: Clearance; IVGTT: Intravenous glucose tolerance test; Vd: Volume of distribution; T½: Half-life.

## Competing interests

The authors declare that they have no competing interests.

## Authors’ contributions

RH provided the study idea, made the calculations, and wrote the manuscript. SL wrote the Ethics application and performed the experiments, which were organized and supervised by TN. All authors read and approved the final manuscript.

## Author information

Robert Hahn is Director at the Research Department at Södertälje Hospital and Professor of Anaesthesia at Linköping University Hospital.

Stefan Ljunggren is specialist in orthopedic surgery and PhD student at Karolinska Institutet.

Thomas Nyström is Associate professor at the Karolinska Institutet, Department of Clinical Science and Education, Södersjukhuset, Section of Internal Medicine, Södersjukhuset, Stockholm.
